# Manipulating Sirtuin 3 pathway ameliorates renal damage in experimental diabetes

**DOI:** 10.1038/s41598-020-65423-0

**Published:** 2020-05-21

**Authors:** Monica Locatelli, Carlamaria Zoja, Cristina Zanchi, Daniela Corna, Sebastian Villa, Silvia Bolognini, Rubina Novelli, Luca Perico, Giuseppe Remuzzi, Ariela Benigni, Paola Cassis

**Affiliations:** 1 Istituto di Ricerche Farmacologiche Mario Negri IRCCS, Centro Anna Maria Astori, Science and Technology Park Kilometro Rosso, Bergamo, Italy; 2 Department of Biomedical and Clinical Sciences, University of Milan, Milan, Italy

**Keywords:** Cell biology, Molecular biology, Kidney diseases

## Abstract

More effective treatments for diabetic nephropathy remain a major unmet clinical need. Increased oxidative stress is one of the most important pathological mechanisms that lead to kidney damage and functional impairment induced by diabetes. Sirtuin 3 (SIRT3) is the main mitochondrial deacetylase and critically regulates cellular reactive oxygen species (ROS) production and detoxification. Honokiol is a natural biphenolic compound that, by activating mitochondrial SIRT3, can carry out anti-oxidant, anti-inflammatory and anti-fibrotic activities. Here, we sought to investigate the renoprotective effects of honokiol in BTBR *ob/ob* mice with type 2 diabetes. Diabetic mice were treated with vehicle or honokiol between the ages of 8 and 14 weeks. Wild-type mice served as controls. Renal *Sirt3* expression was significantly reduced in BTBR *ob/ob* mice, and this was associated with a reduction in its activity and increased ROS levels. Selective activation of SIRT3 through honokiol administration translated into the attenuation of albuminuria, amelioration of glomerular damage, and a reduction in podocyte injury. SIRT3 activation preserved mitochondrial wellness through the activation of SOD2 and the restoration of PGC-1α expression in glomerular cells. Additionally, the protective role of SIRT3 in glomerular changes was associated with enhanced tubular *Sirt3* expression and upregulated renal *Nampt* levels, indicating a possible tubule-glomerulus retrograde interplay, which resulted in improved glomerular SIRT3 activity. Our results demonstrate the hitherto unknown renoprotective effect of SIRT3 against diabetic glomerular disease and suggest that the pharmacological modulation of SIRT3 activity is a possible novel approach to treating diabetic nephropathy.

## Introduction

There are almost 425 million diabetes patients worldwide^1^. Diabetic nephropathy (DN) is one of the main complications associated with diabetes. It affects one-third of diabetic patients and is the largest cause of end-stage renal disease (ESRD) in the Western world^2^. Blood pressure and sugar control, and the blockade of the Renin-Angiotensin-System (RAS) remain the mainstays of therapy for patients with DN^3^. However, the incomplete renoprotective effects that current pharmacological interventions may provide - particularly when started at advanced stages of the disease^4^ - create the need for more effective therapeutic approaches.

One known risk factor for and significant mechanistic contributor to the development of DN is oxidative stress^5^. It derives from an imbalance between increased production of reactive oxygen species (ROS) and insufficient antioxidant capacity of the cell, and is a common feature of all cells that are damaged by diabetes-related hyperglycemia^6^. Excessive ROS production is associated with, among other factors, the inactivation of antioxidant enzymes, such as superoxide dismutase 2 (SOD2)^7^, and mitochondrial dysfunction. Indeed, mitochondria are the main source of intracellular ROS, and changes in mitochondrial morphology and dynamics under hyperglycemic conditions contribute to increased mitochondrial ROS levels^8,9^.

In mitochondria, Sirtuin 3 (SIRT3) is the major NAD^+^-dependent deacetylase^10^ that controls a plethora of processes, including antioxidant pathway and energy metabolism^11–14^. In line with these findings, several studies have highlighted the critical role that SIRT3 has in regulating mitochondrial homeostasis in both healthy and diseased kidneys^15^. As for diabetes, it has been shown that, in kidney biopsies from patients with diabetic nephropathy, *Sirt3* mRNA expression is downregulated and treatments that aim to reduce oxidative stress are renoprotective in mice with DN^16^. However, whether and to what extent treatments that can directly activate SIRT3 are beneficial in DN remains unknown. Recently, it has been demonstrated that honokiol, a major bioactive compound isolated from magnolia bark, effectively limited podocyte damage and the progression of hypertensive nephropathy by activating SIRT3 signaling in mice that were receiving chronic intraperitoneal injections of angiotensin II^17^.

Based on the above evidence, we sought here to evaluate whether SIRT3 activation through honokiol can confer protection against renal disease progression in BTBR *ob/ob* mice, which reproduce key features of human DN better than most murine models type 2 DN^18–20^, starting the treatment at an established phase of the disease to better mimic the condition in humans.

## Results

### SIRT3 is reduced in experimental diabetes, associates with increased ROS levels and is normalized by honokiol

**Figure 1:**
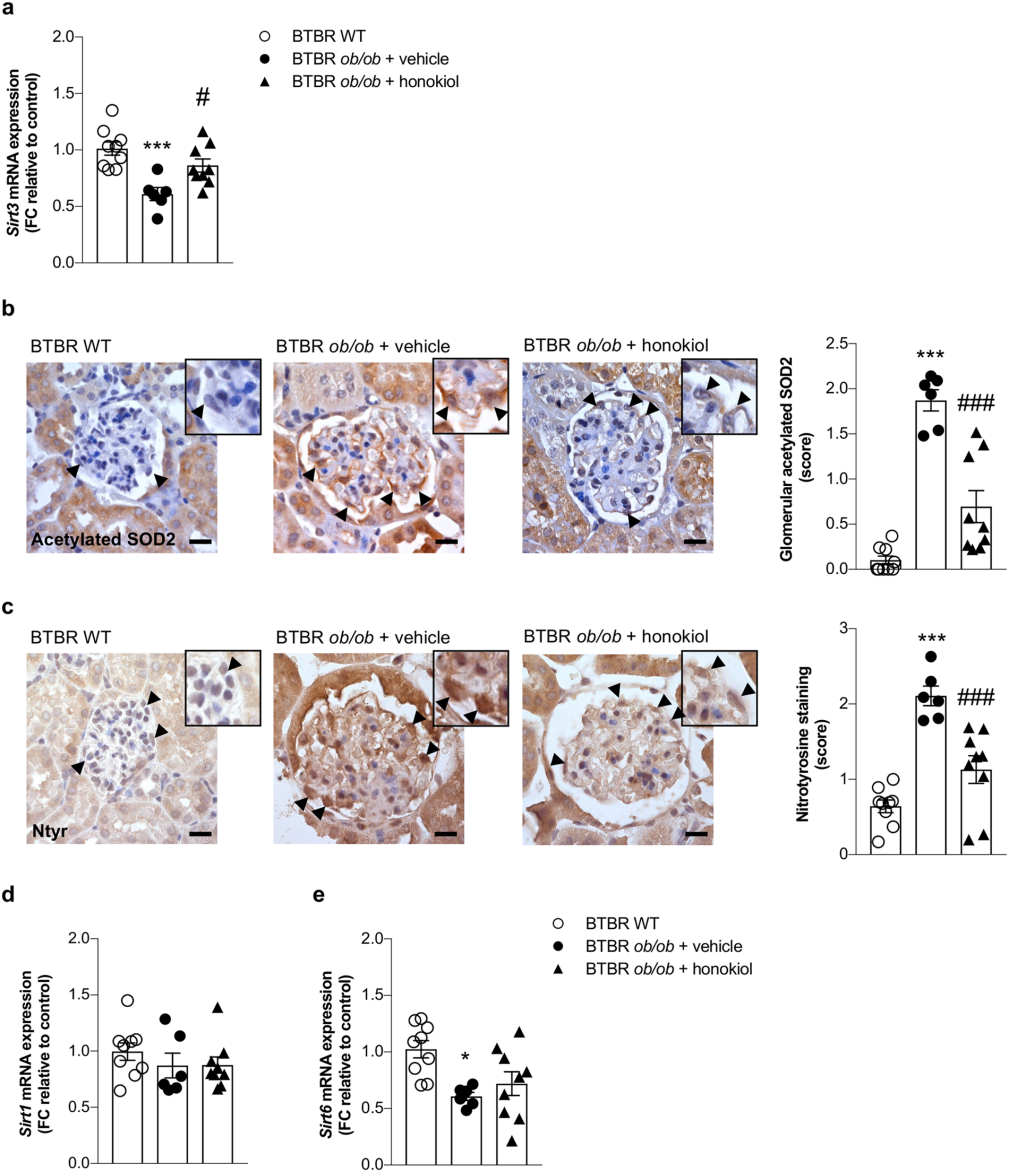
Reduction of renal expression and activity of *Sirt3* paralleled with an increase of ROS production in diabetic mice. (**a**) qRT-PCR analysis of *Sirt3* mRNA levels at 14 weeks of age in kidney of BTBR wild-type (WT) mice (n = 9) and BTBR *ob/ob* mice treated with vehicle (n = 6) or honokiol (n = 9) from 8 weeks of age. (**b**) Representative images and quantification of glomerular acetylated SOD2 staining in BTBR WT mice (n = 9) and in BTBR *ob/ob* mice treated with vehicle (n = 6) or honokiol (n = 9) at 14 weeks of age. Insets show acetylated SOD2 in podocytes of WT and diabetic mice (arrowheads). Scale bars: 20 μm. (**c**) Representative images and quantification of glomerular nitrotyrosine staining in BTBR WT mice (n = 9) and in BTBR *ob/ob* mice treated with vehicle (n = 6) or honokiol (n = 9) at 14 weeks of age. Insets show nitrotyrosine staining in podocytes of WT and diabetic mice (arrowheads). Scale bars: 20 μm. (**d,e**) qRT-PCR analysis of *Sirt1* (**d**), and *Sirt6* (**e**) mRNA levels in kidney of BTBR WT mice (n = 9) and BTBR *ob/ob* mice treated with vehicle (n = 6) or honokiol (n = 9) at 14 weeks of age. Data are mean ± SEM and were analyzed by one-way ANOVA followed by Tukey’s multiple comparisons test, *p < 0.05, ***p < 0.001 vs BTBR WT mice; ^#^*p* < 0.05, ^###^*p* < 0.001 vs BTBR *ob*/*ob* + vehicle.

To investigate whether SIRT3 plays a role in the pathogenesis of DN, we analyzed the gene expression of *Sirt3* in the kidneys of BTBR WT and diabetic BTBR *ob/ob* mice. RT-PCR analysis showed that renal *Sirt3* mRNA expression was markedly lower in BTBR *ob/ob* mice than that observed in WT mice (*p* < 0.001, Fig. 1a). Given that SOD2 is a major target of SIRT3 deacetylase activity, we measured the glomerular expression of acetylated SOD2 at Lysine 68, the specific residue regulated by SIRT3^21^. A greater expression of acetylated-SOD2 was detected in the glomeruli of BTBR *ob/ob* mice than in those of WT mice (*p* < 0.001), indicating lower SIRT3 deacetylase activity in the diabetic mice (Fig. 1b). If reduced SIRT3 activity is actually responsible for the inactivation of SOD2, we expected these changes to be paralleled by an increase in renal oxidative stress in diabetic mice. Indeed, immunohistochemical analysis showed that nitrotyrosine staining – a marker of peroxynitrite‐mediated oxidative damage – was upregulated significantly in the glomerular cells of BTBR *ob/ob* mice compared to those of WT mice (Fig. 1c).

At this point we wondered whether the manipulation of the SIRT3 pathway with honokiol could have a renoprotective effect. Treatment with honokiol restored *Sirt3* expression in BTBR *ob/ob* mice to levels comparable to BTBR WT mice (*p* < 0.05 versus vehicle, Fig. 1a). This effect was associated with a significant reduction in SOD2 acetylation in the glomeruli of treated BTBR *ob/ob* mice (*p* < 0.001 versus vehicle, Fig. 1b) and in nitrotyrosine expression (*p* < 0.001 versus vehicle, Fig. 1c). To rule out the possibility that the protective effect is mediated by SIRT3 and not shared by other sirtuins, we evaluated the expression of *Sirt1* and *Sirt6*, as these are the most involved in renal disorders^15^. We found that the renal expression of *Sirt1* was unchanged in diabetic mice (Fig. 1d) while *Sirt6* was downregulated (Fig. 1e). Honokiol did not affect either *Sirt1* or *Sirt6* expression (Fig. 1d,e) suggesting selective activation of SIRT3.

### Honokiol attenuates albuminuria and ameliorates glomerular injury

**Figure 2:**
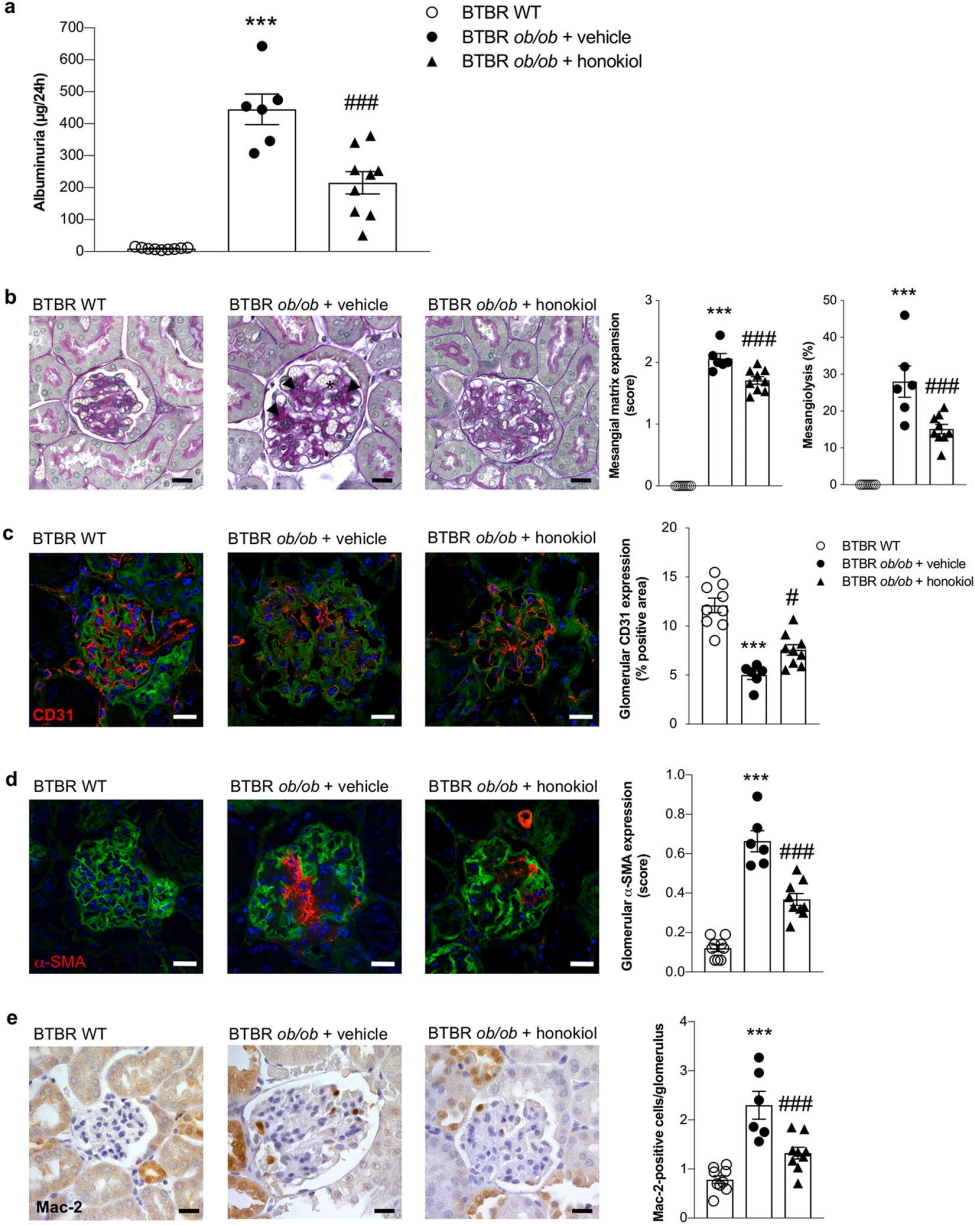
SIRT3 activation through honokiol treatment limits albuminuria, glomerular structural lesions and inflammation in diabetic mice. (**a**) Urinary albumin excretion at 14 weeks of age in BTBR WT mice (n = 9) and BTBR *ob/ob* mice treated with vehicle (n = 6) or honokiol (n = 9). (**b**) Periodic acid-Schiff-stained sections of representative glomeruli and quantification from BTBR WT mice (n = 9) and from diabetic mice treated with vehicle (n = 6) or honokiol (n = 9) at 14 weeks of age showing glomerular lesions, consisting of mesangial matrix expansion (arrowheads) and mesangiolysis (asterisk). Scale bars: 20 μm. (**c,d**) Representative images and quantification of glomerular CD31 (**c**) and α-SMA expressions (**d**) in BTBR WT mice (n = 9) and in BTBR *ob/ob* mice treated with vehicle (n = 6) or honokiol (n = 9) at 14 weeks of age. Nuclei were stained with DAPI and renal structure with lectin FITC-wheat germ agglutinin (WGA). Scale bars: 20 μm. (**e**) Representative images and quantification of glomerular accumulation of Mac-2-positive monocytes/macrophages in BTBR WT mice (n = 9) and in BTBR *ob/ob* mice treated with vehicle (n = 6) or honokiol (n = 9) at 14 weeks of age. Scale bars: 20 μm. Data are mean ± SEM and were analyzed by one-way ANOVA followed by Tukey’s multiple comparisons test, ***p < 0.001 vs BTBR WT mice; ^#^*p* < 0.05, ^###^*p* < 0.001 vs BTBR *ob/ob* + vehicle.

To further examine the renoprotective effect of SIRT3 activation through honokiol treatment, we measured urinary albumin excretion and analyzed the histologic lesions in the kidney of BTBR *ob/ob* mice receiving vehicle or honokiol. We observed that 14-week-old vehicle BTBR *ob/ob* mice exhibited significantly higher levels of albuminuria than BTBR WT mice of the same age (*p* < 0.001). BTBR *ob/ob* mice treated with honokiol had a significantly lower albuminuria levels (*p* < 0.001) compared to mice receiving vehicle (Fig. 2a).

Diabetic BTBR *ob/ob* mice developed renal lesions consisting of mesangial matrix accumulation accompanied by mesangiolysis, resulting in disrupted sites where capillary loops are anchored into the mesangium, which leads to the dilation of the capillary loop (Fig. 2b). In association with mesangial matrix accumulation, the glomeruli of BTBR *ob/ob* mice on vehicle exhibited a glomerular capillary rarefaction (*p* < 0.001 versus WT mice), as revealed by immunofluorescence for CD31, a specific endothelial marker (Fig. 2c), and an increase in α-smooth muscle actin (α-SMA) expression (*p* < 0.001 versus WT mice), a marker of mesangial cell activation (Fig. 2d). Tubular damage in BTBR *ob/ob* mice was mild, as reported previously^18–20^. Honokiol treatment significantly (*p* < 0.001) limited mesangial matrix expansion and mesangiolysis (Fig. 2b). As shown in Fig. 2c, honokiol limited the decrease in CD31 staining (*p* < 0.05 versus BTBR *ob/ob* + vehicle), and glomerular α-SMA expression was also significantly (*p* < 0.001) lower in BTBR *ob/ob* mice after honokiol compared with vehicle (Fig. 2d).

Since recent studies have highlighted the critical role that SIRT3 plays in regulating the inflammatory response^22–24^, we investigated whether SIRT3-activating treatment with honokiol could reduce inflammation in diabetic mice. BTBR *ob/ob* mice that received vehicle exhibited a significantly greater accumulation of Mac-2-positive monocytes/macrophages at the glomerular level compared with BTBR WT mice (*p* < 0.001, Fig. 2e), and honokiol effectively reduced the number of glomerular Mac-2-positive infiltrating cells (*p* < 0.001 versus vehicle, Fig. 2e).

Honokiol did not affect major systemic alterations that had occurred in diabetic mice, such as increased body weight, hyperglycemia and dyslipidemia (Table 1). On the contrary, kidney weight, which had increased significantly in BTBR *ob/ob* mice receiving vehicle (*p* < 0.01 versus BTBR WT mice), was significantly lower after honokiol treatment (*p* < 0.05 versus vehicle) (Table 1).

**Table 1:** Systemic and laboratory parameters in BTBR WT mice and BTBR *ob/ob* mice at 14 weeks of age.

Group	Body weight	Blood glucose	Plasma cholesterol	Plasma triglycerides	Kidney weight
	(g)	(mg/dl)	(mg/dl)	(mg/dl)	(g)
BTBR WT (n = 9)	37 ± 1	131 ± 4	133 ± 3	117 ± 5	0.58 ± 0.02
BTBR *ob/ob*					
vehicle (n = 6)	49 ± 4***	569 ± 20***	155 ± 8*	226 ± 15***	0.95 ± 0.10***
honokiol (n = 9)	49 ± 1***	559 ± 15***	150 ± 4*	211 ± 15***	0.71 ± 0.05^#^

Data are expressed as mean ± SEM. *p < 0.05, ***p < 0.001 vs BTBR WT mice; ^#^*p* < 0.05 vs BTBR *ob/ob* + vehicle.

By the end of the study period, the following mortality was recorded for the BTBR *ob*/*ob* mice: six out of twelve mice in the vehicle group died, while only three out of twelve mice in the honokiol group died. All BTBR WT control mice were alive.

### Honokiol limits podocyte injury in diabetic mice

**Figure 3:**
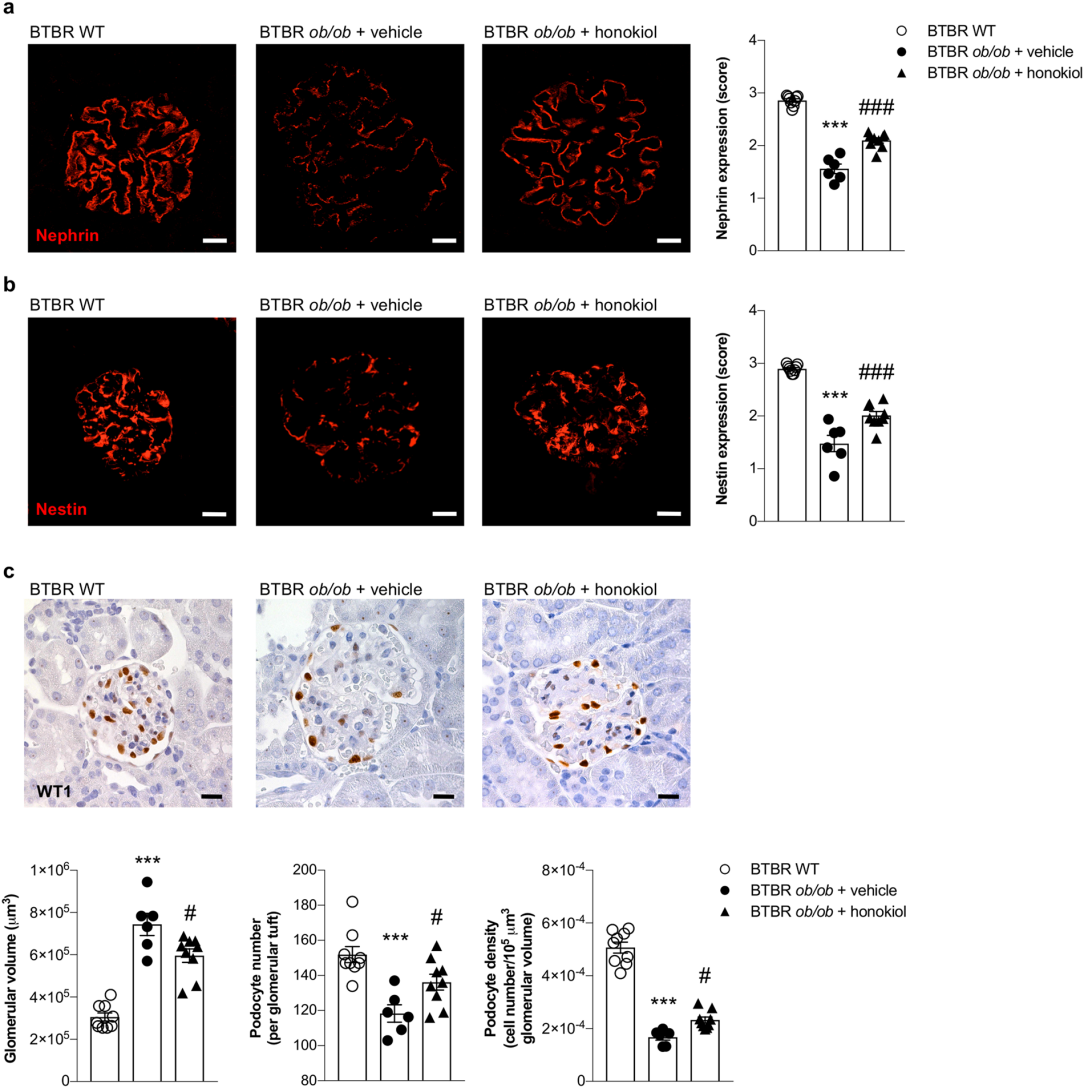
Increase of SIRT3 counteracts podocyte damage and loss in BTBR *ob/ob* mice. (**a,b**) Representative images and quantification of glomerular nephrin (**a**) and nestin (**b**) expressions (red) in BTBR WT mice (n = 9) and in BTBR *ob/ob* mice treated with vehicle (n = 6) or honokiol (n = 9) at 14 weeks of age. (**c**) Representative images and morphometric analysis of glomerular volume, WT1-positive podocytes, expressed as number per glomerulus, and podocyte density in BTBR WT mice (n = 9) and in BTBR *ob/ob* mice treated with vehicle (n = 6) and honokiol (n = 9) at 14 weeks of age. Scale bars: 20 μm. Data are mean ± SEM and were analyzed by one-way ANOVA followed by Tukey’s multiple comparisons test, ***p < 0.001 vs BTBR WT mice; ^#^*p* < 0.05, ^###^*p* < 0.001 vs BTBR *ob/ob* + vehicle.

To clarify what the cellular basis is for the observed reduction in albuminuria achieved by using honokiol in BTBR *ob/ob* mice, we examined the expression of nephrin - an essential component of the podocyte slit diaphragm that is crucial for maintaining slit pore integrity and renal filtration capacity^25^ - and of nestin - a podocyte cytoplasmic protein that is involved in the organization of the cellular cytoskeleton and which plays an important role in maintaining normal podocyte function^26^. Immunofluorescence analyses showed intense nephrin and nestin signals with the typical epithelial-like staining pattern in the glomeruli of WT mice, while BTBR *ob/ob* mice receiving vehicle exhibited a consistent reduction and altered expression pattern for both markers (*p* < 0.001 versus WT mice, Fig. 3a,b). Honokiol treatment ameliorated the defective nephrin and nestin expression and pattern in diabetic mice (*p* < 0.001 versus vehicle Fig. 3a,b).

We then evaluated glomerular volume and podocyte number and density. Morphometric analysis of kidney sections revealed that glomerular volume increased in BTBR *ob/ob* mice that received vehicle (*p* < 0.001 versus WT mice, Fig. 3c). The number of WT1-positive podocytes per glomerulus was lower than in WT mice (*p* < 0.001), which translated into a significant reduction in podocyte density (*p* < 0.001, Fig. 3c). Honokiol reduced glomerular hypertrophy (*p* < 0.05 versus vehicle, Fig. 3c) and limited podocyte depletion (*p* < 0.05 versus vehicle, Fig. 3c).

### Honokiol maintains mitochondrial homeostasis in the glomeruli of BTBR *ob/ob* mice

**Figure 4:**
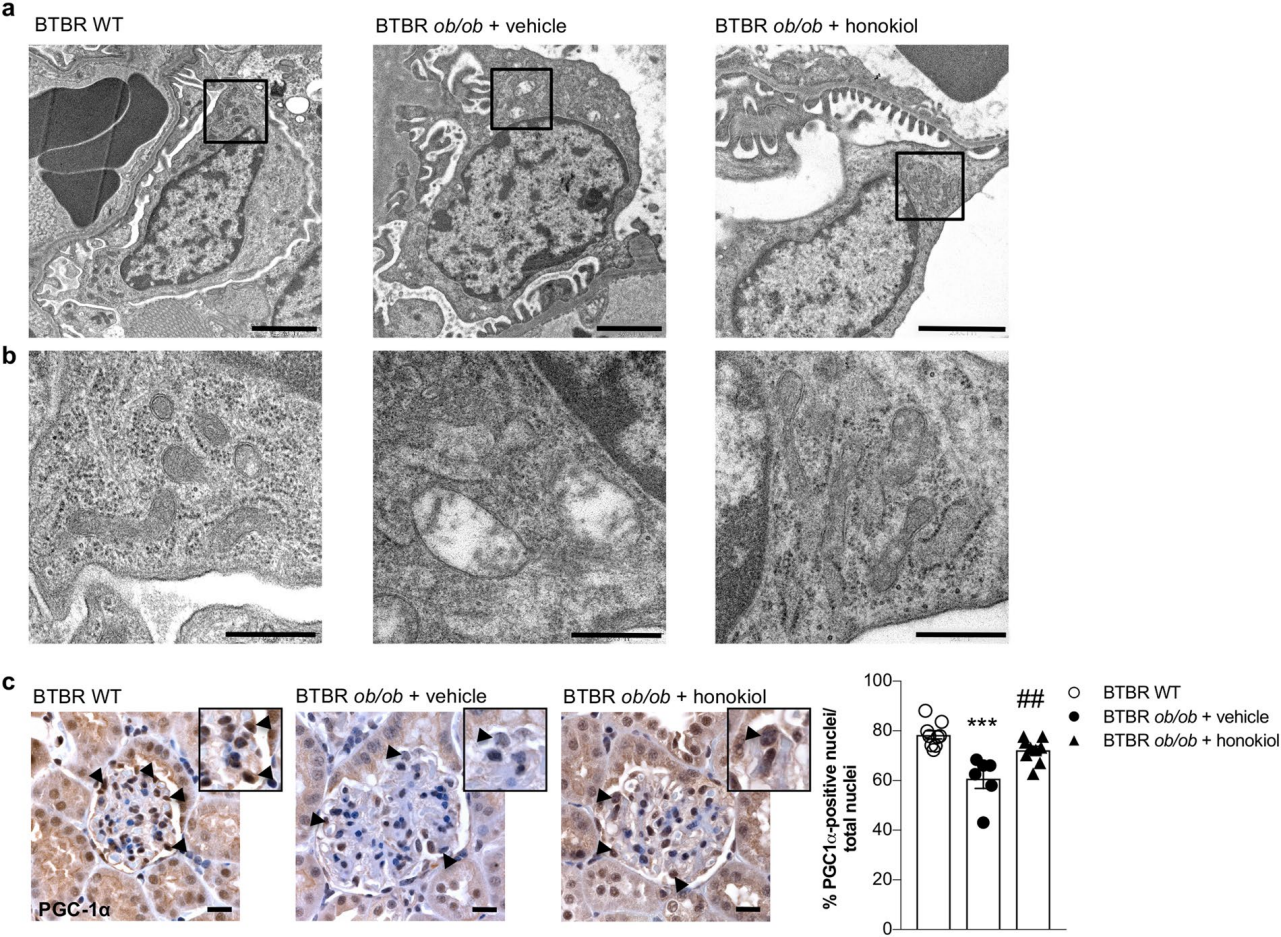
Modulation of SIRT3 preserves mitochondria ultrastructure and homeostasis in podocytes. (**a**) Representative electron microscopy images of podocytes from BTBR WT mice and from BTBR *ob/ob* mice treated with vehicle or honokiol at 14 weeks of age. Scale bars: 2 μm. (**b**) Enlarged images show mitochondria structure in podocytes of WT and diabetic mice. Scale bars: 500 nm. (**c**) Representative images and quantification of glomerular PGC-1α expression in BTBR WT mice (n = 9) and in BTBR *ob/ob* mice treated with vehicle (n = 6) and honokiol (n = 9) at 14 weeks of age. Honokiol increases the percentage of PGC-1α-positive nuclei in diabetic mice (arrowheads and insets). Scale bars: 20 μm. Data are mean ± SEM and were analyzed by one-way ANOVA followed by Tukey’s multiple comparisons test, ***p < 0.001 vs BTBR WT mice; ^##^*p* < 0.01 vs BTBR *ob/ob* + vehicle.

Given the role of SIRT3 in regulating mitochondrial structure and function, we investigated whether SIRT3 activation through honokiol translated into improvements in mitochondrial wellness. We therefore analyzed mitochondrial morphology using transmission electron microscopy. Podocytes of BTBR *ob/ob* mice given vehicle exhibited mitochondrial swelling that was associated with disarrangement of cristae, in contrast with the podocytes of WT mice with normal mitochondria morphology (Fig. 4a,b). Honokiol treatment reversed mitochondria abnormalities induced by diabetes (Fig. 4a,b).

To further assess the protective role of honokiol treatment, we investigated the expression of peroxisome proliferator-activated receptor gamma coactivator 1-alpha (PGC-1α) – the master regulator of mitochondrial function^27,28^. We found that PGC-1α was significantly lower in the glomerular cells of diabetic mice (*p* < 0.001), while its expression was restored to control levels through treatment with honokiol (*p* < 0.01 versus vehicle, Fig. 4c).

### Honokiol restores tubular expression of Sirt3

**Figure 5:**
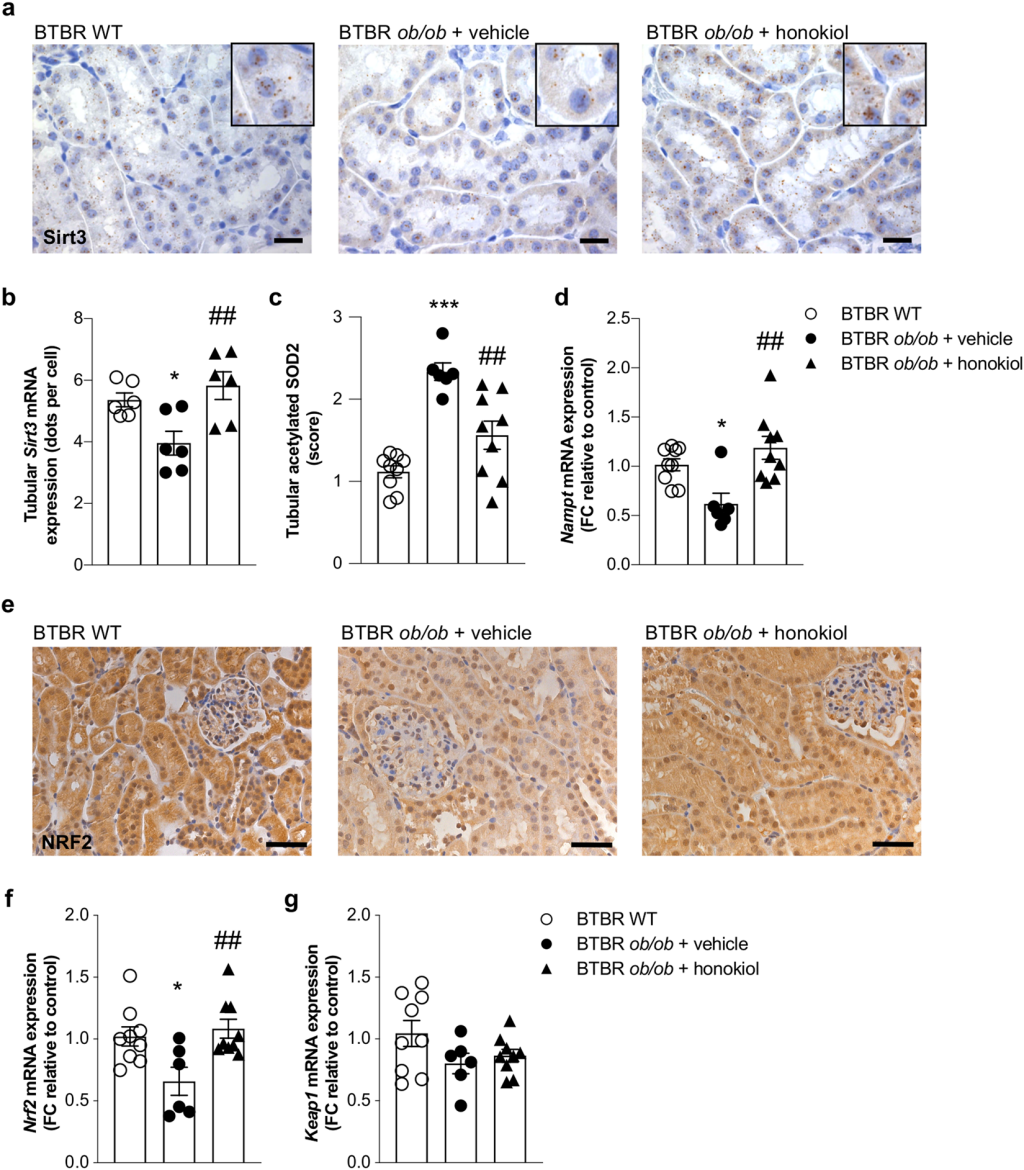
Glomerular SIRT3 activation is associated with enhanced tubular *Sirt3* expression. (**a**,**b**) Representative images (**a**) and quantification (**b**) of *in situ* hybridization for *Sirt3* in kidney cortex from BTBR WT mice and BTBR *ob/ob* mice treated with vehicle or honokiol at 14 weeks of age. Scale bars: 20 μm. Insets show the localization of *Sirt3* in tubular cells. Image-based quantitative software analysis was performed to evaluate tubular expression of *Sirt3*. Fiji Image J software (https://imagej.net/Fiji) was used for the quantification of the number of *Sirt3* mRNA dots (representing single mRNA molecules). Orbit Image analysis software (http://orbit.bio) was used to count the number of cells. *Sirt3* mRNA levels were expressed as average number of dots per cell. (**c**) Quantification of tubular acetylated SOD2 staining in BTBR WT mice (n = 9) and in BTBR *ob/ob* mice treated with vehicle (n = 6) or honokiol (n = 9) at 14 weeks of age. (**d**) qRT-PCR analysis of *Nampt* mRNA levels in kidney of BTBR WT mice (n = 9) and BTBR *ob/ob* mice treated with vehicle (n = 6) or honokiol (n = 9). (**e**) Representative images of NRF2 expression in BTBR WT mice (n = 9) and in BTBR *ob/ob* mice treated with vehicle (n = 6) or honokiol (n = 9) at 14 weeks of age. Scale bars: 50 μm. (**f,g**) qRT-PCR analysis of *Nrf2* (**f**) and *Keap1* (**g**) mRNA levels in kidney of BTBR WT mice (n = 9) and BTBR *ob/ob* mice treated with vehicle (n = 6) or honokiol (n = 9). Data are mean ± SEM and were analyzed by one-way ANOVA followed by Tukey’s multiple comparisons test, *p < 0.05, ***p < 0.001 vs BTBR WT mice; ^##^*p* < 0.01 vs BTBR *ob/ob* ^+^ vehicle.

The modulation of total *Sirt3* mRNA expression and its upregulation by honokiol in diabetic mice prompted us to assess *Sirt3* mRNA localization in the kidney through *in situ* hybridization experiments. We found that *Sirt3* was predominantly expressed in the tubular compartment of WT mice (Fig. 5a). In BTBR *ob/ob* mice given vehicle, the tubular expression of *Sirt3* was significantly lower compared with WT mice (*p* < 0.05), but it was restored (*p* < 0.01 versus vehicle) through honokiol treatment (Fig. 5a,b). In contrast, a weak *Sirt3* mRNA signal was found in the glomeruli of WT mice, which was not altered in either diabetic mice given vehicle or in those receiving honokiol (data not shown). To test whether increased *Sirt3* mRNA levels, after honokiol treatment, were associated with its increased activity, we analyzed SOD2 acetylation status also in the tubules. Similar to the glomerular compartment, we observed an increased acetylation of SOD2 in the proximal tubules of BTBR *ob/ob* mice given vehicle, which was reduced in mice after honokiol treatment (*p* < 0.01 *vs* vehicle, Fig. 5c). To investigate whether the increased expression and activity of *Sirt3* in tubular cells of honokiol-treated diabetic mice can contribute also to increased SIRT3 activity in the glomerular compartment, as it has been established with regard to SIRT1^29^, we evaluated the expression of nicotinamide phosphoribosyltransferase (NAMPT), the rate-limiting enzyme for the biosynthesis of nicotinamide adenine dinucleotide (NAD^+^), the essential co-factor of SIRT3 activity. Our results showed that *Nampt* mRNA expression was significantly lower in the kidneys of BTBR *ob/ob* mice given vehicle than in those of WT mice (*p* < 0.05), and treatment with honokiol restored *Nampt* levels (*p* < 0.01 versus vehicle) in the diabetic mice (Fig. 5d).

In order to identify the potential mechanism through which honokiol treatment could promote SIRT3 expression and exert anti-oxidant activities, we investigated the expression of nuclear factor erythroid 2-related factor 2 (NRF2), the master regulator of anti-oxidant response^30–32^. As shown in Fig. 5e, we found that NRF2 protein expression was reduced in the nucleus and cytoplasm of BTBR *ob/ob* mice given vehicle compared to BTBR WT mice. Treatment with honokiol restored the expression of NRF2 (Fig. 5e). These data were confirmed by real time RT-PCR experiments that showed a significant reduction of *Nrf2* in BTBR *ob/ob* mice given vehicle compared to BTBR WT mice (Fig. 5f). Treatment with honokiol normalized *Nrf2* expression (Fig. 5f). Conversely, the expression of the NRF2 adaptor protein *Keap1* was unchanged in any of the experimental groups (Fig. 5g), possibly suggesting that KEAP1 is not involved in the maintenance of the homeostatic levels of NRF2.

## Discussion

In this study, we demonstrated that in experimental diabetes 1) renal SIRT3 expression and activity are reduced and are associated with increased oxidative stress; 2) selective manipulation of the SIRT3 pathway using honokiol protects against albuminuria, glomerular lesions and inflammation; 3) honokiol reduces podocyte damage and loss by preserving mitochondrial structure and function.

The first finding of this study is that *Sirt3* mRNA expression was lower in the kidneys of BTBR *ob/ob* mice, which is in line with earlier observations in experimental type 1 diabetes^33^ and in kidney biopsies from DN patients^16^. The reduction in *Sirt3* expression in BTBR *ob/ob* mice was associated with an impairment in its deacetylase activity towards SOD2 and an increase in ROS levels. Oxidative stress is a well-known driver of diabetic kidney disease^5^, and in this regard our study shows that SIRT3-SOD2 axis is a critical modulator of renal oxidative stress in diabetic mice and that this molecular pathway could be targetable through honokiol. Indeed, we observed that honokiol enhanced *Sirt3* mRNA levels in BTBR *ob/ob* mice, and this had a positive impact on SOD2 activity and oxidative stress. Previous studies have elegantly demonstrated that in the heart honokiol can enter mitochondria and physically bind to SIRT3, enhancing its affinity for NAD^+^ with immediate consequences on deacetylase activity and ROS production^34^. However, whether honokiol’s action is exclusively directed towards the activation of SIRT3 and not shared with other sirtuins has not been addressed in these studies. The finding here that honokiol did not modulate the renal expression of either *Sirt1* or *Sirt6*, which are, along with SIRT3, involved in kidney disease progression^15^, clearly demonstrates the beneficial effects of honokiol on the selective restoration of SIRT3 in the kidney.

Improvements in albuminuria and glomerular damage after treatment of BTBR *ob/ob* mice with honokiol appear to be relatively consistent. Furthermore, the large reduction in glomerular inflammation we observed in these mice demonstrates that SIRT3 has a potent anti-inflammatory effect on the diabetic kidney that has never been reported before. Glomerular hypertrophy has been identified as a characteristic feature of diabetic glomerular injury in experimental models and in humans. This is accompanied by the inability of podocytes to maintain complete coverage of the glomerular filtration surface area, which results in podocyte hypertrophic stress, leading to podocyte detachment^35^. Podocytes are critical players in maintaining the integrity of the glomerular filtration barrier, and podocyte injury and loss have been identified as the initial event in the development of proteinuria and glomerular lesions, ending in glomerulosclerosis. The evidence indicates that the podocyte is the earliest target of diabetic injury, and indeed podocyte number correlates with albuminuria, and glomerular podocyte density appears to be one of the best predictors of albuminuria and disease progression^36^. In this study, the anti-albuminuric effect of honokiol was associated with an amelioration of the defective expression of podocyte proteins and a reduction in podocyte loss in BTBR *ob/ob* mice. Increasing evidence indicates that the disruption of mitochondrial bioenergetics in podocytes is crucial for dictating the development and progression of DN^37–39^, and recent findings have provided novel correlations between podocyte mitochondrial morphology and the progression of DN^40,41^. Here, treatment with honokiol improved podocyte mitochondrial abnormalities in diabetic mice and also restored the expression of PGC-1α, a known modulator of mitochondrial homeostasis, thereby reinforcing the hypothesis that increasing SIRT3 activity is crucial in the preservation of mitochondrial integrity^42,43^. According to a recent study^44^, it is conceivable that the ability of honokiol to modulate PGC-1α could be dependent on the activation of the AMPK-CREB pathway by SIRT3, ultimately resulting in PGC-1α stimulation.

To further investigate the mechanisms underlying honokiol-induced SIRT3 activation in DN in greater depth, we analyzed the localization of *Sirt3* expression in the kidneys of BTBR *ob/ob* mice. We observed that *Sirt3* levels in tubular cells were lower in diabetic mice and were restored by honokiol treatment, accompanied by a reduction in the acetylation of the SIRT3 target SOD2. The possible mechanism through which honokiol stimulates SIRT3 protein expression relies on NRF2 that we found increased in the kidney of treated diabetic mice. That NRF2 can upregulate SIRT3 expression is supported by the evidence that NRF2 induces SIRT3 expression through the direct binding of NRF2 subunit to the SIRT3 promoter^45^.

On the other hand, we found that *Sirt3* expression was not modulated by honokiol in the glomeruli of BTBR *ob/ob* mice. These results reminded us of those reported in an interesting study by Hasegawa *et al*.^29^, in which the selective upregulation of tubular SIRT1 mediated a retrograde interplay between tubules and podocytes, which resulted in the amelioration of diabetes-associated glomerular disease. In that study, the authors reported that the upregulation of NAMPT in tubular cells, mediated by increased SIRT1 levels, maintained adequate nicotinamide mononucleotide (NMN) concentrations around glomeruli to promote podocyte function, ultimately translating into an anti-albuminuric effect in diabetes. NAMPT is also a critical regulator of SIRT3 activity, as it produces NMN, the intermediate product for NAD^+^ biosynthesis^46,47^. Consistent with the interpretation provided by Hasegawa^29^ we suggest that the protective effect of SIRT3 on albuminuria and glomerular changes in BTBR *ob/ob* mice could be due to tubule-glomerulus retrograde interplay. In particular, the upregulation of SIRT3 and NAMPT in tubular cells may provide the required amount of NMN to diabetic podocytes and other glomerular cells, ultimately supplying glomerular NAD^+^ to further increase SIRT3 activity in a virtuous cycle.

In conclusion, here we have shown that SIRT3 plays a crucial role in the pathogenesis of DN and that its specific activation through honokiol reduces diabetes-induced oxidative stress and protects podocytes and, generally, the glomerulus from diabetes-induced damage. In the context of the great interest that antioxidant therapies have generated, as promising treatments for DN and other chronic diabetic complications^48^, our data provide evidence of a hitherto unknown protective effect of SIRT3 against diabetic glomerular disease. This suggests that the pharmacological activation of SIRT3 activity may be an effective, novel approach for treating DN.

## Methods

### Experimental design

Male BTBR (black and tan, brachyuric) Lep^*ob/ob*^ and BTBR wild-type (WT) mice were obtained from Jackson Laboratories (Bar Harbor, ME, USA) and were kept on specific pathogen-free facility with constant temperature on a 12:12-hour light-dark cycle with free access to standard diet and water. At 8 weeks of age, when they had already developed albuminuria, BTBR *ob/ob* mice were randomly allocated to receive (n = 12 mice/group): vehicle (DMSO in saline solution) or honokiol (10 mg/kg in DMSO; BioVision, Milpitas, CA, USA) by once-daily intraperitoneal injection. The dose of honokiol was chosen according to available data in the literature^49^. Treatment lasted until mice were 14 weeks old. BTBR WT mice (n = 9) were followed for the same length of time as controls. Mice were euthanized through CO_2_ inhalation and their kidneys were collected and processed for analysis. Before sacrifice, mice were housed in metabolic cages for 24-hour urine collection for albuminuria assessment. Blood samples were collected for glucose, cholesterol and triglyceride measurements. The experimenters were not blind to the treatment, but they were blind for measurement of experimental outcomes. All animal experiments were conducted in accordance with institutional guidelines in compliance with national (D.L.n.26, March 4, 2014), and international laws and policies (directive 2010/63/EU on the protection of animals used for scientific purposes) and were approved by the Institutional Animal Care and Use Committees of Istituto di Ricerche Farmacologiche Mario Negri IRCCS.

### Biochemical parameters

Biochemical parameters were assessed as we previously described^20,50^. Blood glucose levels were assessed with a reflectance meter (OneTouch UltraEasy, LifeScan, Milpitas, CA, USA). Plasma cholesterol and triglycerides were measured using the Reflotron test (catalog 10745065202 and catalog 10745049202, Roche Diagnostic Corporation, Indianapolis, IN, USA). Urinary albumin excretion was measured with the ELISA test using the Bethyl test kit (catalog E101, catalog A90-134A and catalog A90-134P, Bethyl Laboratories Inc, Montgomery, TX, USA).

### Quantitative (q) RT-PCR

Total RNA was isolated from whole kidney tissue as we previously described^51^. Briefly, TRIzol Reagent (catalog 15596026, Thermo Fisher Scientific, Waltham, MA, USA) was used according to the manufacturer’s instructions. After treatment with DNase (catalog 6101, Promega Madison, WI, USA), cDNA was prepared using the SuperScript VILO cDNA synthesis kit (catalog 11754050, Thermo Fisher Scientific). qRT-PCR analyses were performed on ABI ViiA 7 Real-Time PCR system (Thermo Fisher Scientific). *Sirt3, Sirt1*, *Sirt6* and *Nampt* were assessed using TaqMan Gene Expression Master Mix (catalog 4369016, Thermo Fisher Scientific) and the following TaqMan assays (Thermo Fisher Scientific): Mm01275638_m1, Mm01168521_m1, Mm01149042_m1 and Mm01293560_m1, respectively. Mouse ACTB Endogenous Control (VIC/MGB probe) was used to evaluate the housekeeping gene *β-actin*. Gene expression levels of *Sirt3, Sirt1*, *Sirt6* and *Nampt* were normalized to *β-actin* levels. *Nrf2* and *Keap1* were assessed using SYBR Green PCR Master Mix (catalog 4367659, Thermo Fisher Scientific) and the following primers (300 nM): *Nrf2* forward 5′-CCCAGCAGGACATGGATTTGA-3′ and reverse 5′-CATAGTCCTTCTGTCGCTGACT-3′; *Keap1* forward 5′-ACGTCCTCGGAGGCTATGAT-3′ and reverse 5′- GGGTCACCTCACTCCAGGTA-3′ *β-actin* (forward 5′-CACTGTCGAGTCGCGTCC-3′ and reverse 5′-TCATCCATGGCGAACTGGTG -3′) was used as endogenous control. Relative quantities were calculated by the 2^-ΔΔCt^ method. Data are presented as fold change relative to WT control group.

### Renal histology

Kidney samples were fixed in Duboscq-Brazil (catalog P0094, Diapath, Bergamo, Italy), dehydrated, and embedded in paraffin, as we previously described^50^. Three-micrometer sections were stained with periodic acid-Schiff (PAS) reagent, and at least 50 glomeruli were examined per animal. The degree of glomerular mesangial matrix expansion was quantified using a score between 0 and 3 (0 = no mesangial matrix expansion; 1 = minimal; 2 = moderate; 3 = diffuse mesangial matrix expansion). The number of glomeruli exhibiting mesangiolysis in an entire kidney section was counted and expressed as a percentage. All biopsies were reviewed by a blinded pathologist. Samples were examined using ApoTome Axio Imager Z2 (Zeiss, Jena, Germany).

### Immunohistochemistry

For immunoperoxidase experiments, as we previously performed^50^, formalin-fixed, 3-μm paraffin-embedded kidney sections were incubated with Peroxidazed 1 (catalog PX968H, Biocare Medical, Pacheco, CA, USA) to quench endogenous peroxidase, after antigen retrieval in a decloaking chamber with DIVA, Rodent or Borg decloaker buffer (catalog DV2004MX, catalog RD913M and catalog BD1000MM, Biocare Medical) to increase the reactivity of antibodies to antigens. After blocking for 30 minutes with Rodent Block M (catalog RBM961G, Biocare Medical), sections were incubated with rabbit anti-SOD2/MnSOD (acetyl K68) (catalog ab137037, Abcam, Cambridge, UK, 1:50 and 1:200), rabbit anti-nitrotyrosine (catalog 06-284, Merck Millipore, Burlington, MA, USA, 1:100), rat anti-Mac 2 (clone M3/38, Cedarlane, Burlington, ON, Canada, 1:600), rabbit anti-PGC-1α (catalog ab54481, Abcam, 1:100) and rabbit anti-NRF2 (catalog ab31163, Abcam, 1:100) antibodies, followed by Rat on Mouse HRP-Polymer or Rabbit on Rodent HRP-Polymer (catalog RT517 and catalog RMR622G, Biocare Medical) for 30 minutes at room temperature. Stainings were visualized using diaminobenzidine (catalog BDB2004H, Biocare Medical) substrate solutions. Slides were counterstained with Mayer’s hematoxylin (catalog MHS80-2.5 L, Bio Optica, Milan, Italy), mounted with Eukitt mounting medium (catalog 09-00250, Bio Optica) and finally observed using light microscopy (ApoTome, Axio Imager Z2, Zeiss). Negative controls were obtained by omitting the primary antibody on adjacent sections. Glomerular acetylated SOD2 and nitrotyrosine stainings were quantified with a semiquantitative score between 0 and 3 (0: absent glomerular staining, 1: weak staining in a few glomerular cells, 2: moderate glomerular staining, 3: intense glomerular staining). At least 15–20 glomeruli/section for each animal were randomly analyzed. Tubular acetylated SOD2 was quantified with a semiquantitative score between 0 and 3 in proximal tubules (0: absent staining, 1: weak staining, 2: moderate staining, 3: intense staining). At least 10-15 fields/section for each animal were randomly analyzed. Mac-2-positive monocyte/macrophages within glomeruli were counted in a minimum of 50 glomerular cross-sections and expressed as average number of cells per glomerulus. Glomerular PGC-1α was evaluated by expressing the positive-PGC-1α nuclei as a percentage of the total nuclei per tuft (ImageJ software). At least 15–20 glomeruli/section per animal were randomly analyzed.

OCT-frozen kidney sections were fixed with cold acetone, blocked in 1% bovine serum albumin (BSA) and then incubated with the following primary antibodies: rat anti-mouse CD31 (catalog 550274, BD Pharmingen, San Jose, CA, 1:100), Cy3-conjugated mouse anti- α-smooth muscle actin (α-SMA) (catalog c6198, clone 1A4, Sigma Aldrich, St. Louis, MO, USA, 1:200), goat anti-nephrin (catalog sc-19000, clone N-20, Santa Cruz Biotechnology Inc, Dallas, TX, USA, 1:100) or rat anti-nestin (catalog ab81462, clone 7A3, Abcam, 1:300) followed by appropriate Cy3-conjugated secondary antibodies (Jackson ImmunoResearch Laboratories, Cambridge, UK). For nephrin staining, the antigen retrieval in citrate buffer was performed. Negative controls were obtained by omitting the primary antibody on adjacent sections. Samples were examined using an inverted confocal laser microscope (LSM 510 Meta, Zeiss). Glomerular CD31-positive staining was quantified in 15 glomeruli in each section and expressing the positive glomerular areas as a percentage of the total area (ImageJ software)^20^. As we previously analyzed^20^, α-SMA, nephrin and nestin stainings were quantified by score from 0 to 3 (α-SMA 0–0.5: absent or weak signal, 1: mild, 2: moderate, 3: intense signal; nephrin, 0–0.5: absent or weak and fragmented signal, 1: linear and thin signal, 2: linear signal, 3: linear and intense signal; nestin, 0–0.5: absent or weak staining in a few podocytes, 1: moderate staining in podocytes with altered distribution, 2: moderate podocyte staining, 3: intense podocyte staining). At least 20 glomeruli/section for each animal were randomly analyzed. Immunohistochemical analysis and scoring assays were done by individuals unaware of sample identity.

### Glomerular podocyte count

Formalin-fixed, 3-μm paraffin-embedded kidney sections were incubated with Peroxidazed 1, after antigen retrieval in a decloaking chamber with Rodent decloaker buffer. After blocking for 30 minutes with Rodent Block M, sections were incubated with rabbit anti-WT1 (catalog ab89901, Abcam, 1:600) antibody followed by Rabbit on Rodent HRP-Polymer for 30 minutes at RT. Stainings were visualized using diaminobenzidine substrate solutions. Slides were counterstained with Mayer’s hematoxylin, mounted with Eukitt mounting medium and finally observed using light microscopy (ApoTome, Zeiss). Negative controls were obtained by omitting the primary antibody on adjacent sections. At least 20 glomeruli/section for each animal were randomly acquired. The estimate of the average number of podocytes per glomerulus and the glomerular volume were determined through morphometric analysis, as previously described^52^.

### Ultrastructural analysis

Mitochondrial morphology was observed by transmission electron microscopy, as we previously performed^51^. Fragments of kidney tissue were fixed overnight in 2.5% glutaraldehyde (catalog 340855, Sigma Aldrich, Darmstadt, Germany) in 0.1 M cacodylate buffer (pH 7.4) (catalog 11652, Electron Microscopy Sciences, Hatfield, PA, USA) and washed repeatedly in the same buffer. After postfixation in 1% OsO_4_, specimens were dehydrated through ascending grades of alcohol and embedded in Epon resin. Ultrathin sections were stained with uranyl acetate replacement (catalog 22405, UAR, Electron Microscopy Sciences, Hatfield, PA 19440, USA) and lead citrate (catalog 22410, Electron Microscopy Sciences) and examined using transmission electron microscopy (Fei Morgagni 268D, Philips, Hillsboro, OR, USA).

### *In situ* hybridization

*Sirt3 in situ* hybridization (ISH) was performed on 3-µm formalin-fixed and paraffin-embedded kidney sections using the RNAscope 2.5 HD Brown Assay kit (catalog 321720, ACD Bio-techne, Minneapolis, MN, USA) according to the manufacturer’s instructions (https://acdbio.com/manual-assays-rnascope). Briefly, deparaffinized slides were treated with hydrogen peroxide, heat and protease before hybridization with *Sirt3* target probe (40 °C for 2 hours; HybEZ Oven). *Sirt3* target probe (catalog 300031, ACD Bio-techne, Minneapolis) is designed on mouse *Sirt3*, transcript variant 3, mRNA reference sequence (NM_001177804 at the National Center of Biotechnology Information) and detects all the murine *Sirt3* transcript variants. After hybridization, slides were washed and processed for standard signal amplification steps. Chromogenic detection was performed using 3, 3’-diaminobenzidine (DAB) followed by counterstaining with 50% Mayer’s hematoxylin (Bio Optica, Milan, Italy). Probes against the housekeeping gene PPIB (peptidylprolyl isomerase B) and the bacterial gene DapB were used as positive and negative controls, respectively. *Sirt3* mRNA molecules were visualized as brown, punctate dots. Ten fields of tubule cells and twenty glomeruli for each animal were randomly acquired using bright-field microscopy (ApoTome, Axio Imager Z2, Zeiss). Image-based quantitative software analysis was performed to evaluate tubular and glomerular expression of *Sirt3*. Fiji Image J software (https://imagej.net/Fiji) was used for the quantification of the number of *Sirt3* mRNA dots (representing single mRNA molecules). Orbit Image analysis software (https://orbit.bio) was used to count the number of cells. *Sirt3* mRNA levels were expressed as average number of dots per cell.

### Statistical analysis

Results were expressed as mean ± SEM and in bar chart with individual data points. Data analysis was performed using Graph Pad Prism software (Graph Pad, San Diego, CA, USA). Comparisons were made using one-way ANOVA with Tukey’s multiple comparisons post hoc test, and the statistical significance was defined as a *p* value of <0.05.

## Data Availability

The datasets generated during and/or analysed during the current study are available from the corresponding author on reasonable request.
